# Knockout of beta‐2 microglobulin reduces stem cell‐induced immune rejection and enhances ischaemic hindlimb repair via exosome/miR‐24/Bim pathway

**DOI:** 10.1111/jcmm.14778

**Published:** 2019-11-15

**Authors:** Yuqing Zhang, Yanli Wang, Lianbo Shao, Xiangbin Pan, Chun Liang, Bin Liu, Yu Zhang, Wenping Xie, Bing Yan, Feng Liu, Xi‐yong Yu, Yangxin Li

**Affiliations:** ^1^ Institute for Cardiovascular Science & Department of Cardiovascular Surgery First Affiliated Hospital of Soochow University Suzhou China; ^2^ Department of Cardiac Surgery Fuwai Hospital Beijing China; ^3^ Department of Cardiology Shanghai Changzheng Hospital Second Military Medical University Shanghai China; ^4^ Department of Cardiology Second Hospital of Jilin University Jilin China; ^5^ Guangzhou Medical University Guangzhou China

**Keywords:** exosome, immune rejection, ischaemic hindlimb repair, mesenchymal stem cell, miRNA, survival

## Abstract

Generating universal human umbilical mesenchymal stem cells (UMSCs) without immune rejection is desirable for clinical application. Here we developed an innovative strategy using CRISPR/Cas9 to generate B2M^‐^UMSCs in which human leucocyte antigen (HLA) light chain β2‐microglobulin (B2M) was deleted. The therapeutic potential of B2M^‐^UMSCs was examined in a mouse ischaemic hindlimb model. We show that B2M^‐^UMSCs facilitated perfusion recovery and enhanced running capability, without inducing immune rejection. The beneficial effect was mediated by exosomes. Mechanistically, microRNA (miR) sequencing identified miR‐24 as a major component of the exosomes originating from B2M^‐^UMSCs. We identified Bim as a potential target of miR‐24 through bioinformatics analysis, which was further confirmed by loss‐of‐function and gain‐of‐function approaches. Taken together, our data revealed that knockout of B2M is a convenient and efficient strategy to prevent UMSCs‐induced immune rejection, and it provides a universal clinical‐scale cell source for tissue repair and regeneration without the need for HLA matching in the future.

## 1 INTRODUCTION

Human umbilical mesenchymal stem cell (UMSCs) is a better choice than autologous bone marrow MSCs for stem cell‐based therapy because of better quality control, cost effectiveness and availability.^1^, ^2^ However, the transplanted cells can be rejected if the human leucocyte antigen (HLA) of donor stem cells are not matched to that of the recipient.^3^ Although immune rejection can be treated with immunosuppressive drugs, the severe side effects associated with the treatment are harmful to the patients.^4^

Immune rejection of stem cells is due to the expression of human leucocyte antigen class I molecules (HLA‐I) on the surface of these cells. HLA‐I presents ‘non‐self’ antigens to CD8^+^ T cells which eliminate the transplanted cells through direct cytotoxic effect. Due to the polymorphic nature of the HLA‐I genes, it is often difficult to identify a perfect match between donor and recipient prior to transplantation.^5^, ^6^ HLA‐I is made up of a heavy chain and a light chain, which is also called β_2_‐microglobulin (B2M). HLA‐I structure is disrupted and non‐functional when the B2M gene is deleted.^6^ Although UMSCs do not express class II antigens, the expression of class I antigen is increased when these cells are injected to ischaemic tissue.^7^ To solve this problem, an universal haematopoietic stem cell clone was generated by deleting B2M using CRISPR/Cas9 technology.^8^ However, it is not known whether B2M can be deleted from UMSCs without impeding the therapeutic effects of these cells.

Emerging evidence suggests that much of the therapeutic effects of MSCs are mediated by exosomes which are membrane vesicles carrying many functional molecules including mRNAs, microRNAs (miRNAs) and proteins.^9^, ^10^, ^11^, ^12^, ^13^, ^14^ MSC‐derived exosomes have been shown to improve heart function by inhibiting inflammation and promoting angiogenesis in animal models of myocardial infarction.^15^, ^16^, ^17^, ^18^ It has been shown that MSC‐derived exosomes release multiple miRNAs that promote cell survival.^19^ Based on our sequencing data of exosomal miRNAs, miR‐24 is highly expressed in exosomes.^20^ Others have reported that miR‐24 expression was decreased in the ischaemic border zone of infarcted mouse myocardium.^21^ Transgenic mice that specifically overexpress miR‐24 in the heart exhibited decreased cardiomyocyte apoptosis when subjected to myocardial infarction.^22^ Overexpression of miR‐24 in haematopoietic cells prevented cell death induced by cytokine and serum withdrawal.^23^ MiR‐24 also hampers chemotherapy‐induced apoptosis in breast cancer stem cells.^24^ In contrast, miR‐24 inhibition induces apoptosis in human non‐small cell lung cancer cells.^25^ These studies suggest that miR‐24 plays an important role in regulating apoptosis. However, the role of miR‐24 in exosome‐mediated skeletal muscle repair remains unknown.

In this study, we generated human UMSCs with B2M deletion (B2M^‐^UMSCs) using CRISPR/Cas9 technology and evaluated the effects of B2M^‐^UMSCs on immune rejection and skeletal muscle repair in a mouse model of hindlimb ischaemia. Moreover, we elucidated the signalling pathways mediating the beneficial effects of B2M^‐^UMSCs.

## 2 MATERIALS AND METHODS

### 2.1 Harvest and identification of UMSC exosomes

Human umbilical mesenchymal stem cells (UMSCs; Jiangsu Heze Biotechnology Co., Ltd., China) were cultured in Dulbecco's modified Eagle's medium:F12 containing 20% foetal bovine serum (FBS). The FBS was centrifuged at 110 000 g to remove bovine‐derived exosomes. Exosomes were isolated from UMSC culture medium (48 hours) using a total exosome isolation kit (Life Technology). The culture medium was first centrifuged at 2000 *g* for 30 minutes to get rid of debris and dead cells, and then transferred to a new tube containing 0.5 volumes of the Total Exosome Isolation reagent. The mixture was incubated at 4°C overnight and centrifuged at 10 000 *g* for 1 hour at 4°C. The pellet was re‐suspended in PBS, and the protein concentration was determined using a BCA protein assay kit (Takara).

The morphology of the exosomes was revealed by transmission electron microscopy. The exosomes were attached to aldehyde/sulphate latex beads (4 μm; Molecular Probes; Invitrogen), then incubated with an FITC‐conjugated antibody against CD63 (Abcam), and the expression of exosome marker CD63 was analysed by flow cytometry and Western blot.

### 2.2 Mouse model of unilateral hindlimb ischaemia

A mouse model of unilateral hindlimb ischaemia was set up to explore the effect of UMSCs in tissue repair. All animals were obtained from the Experimental Animal Center of Soochow University. The animal experiments were approved by the Animal Care and Use Committee of Soochow University. We randomly divided 8‐ to 12‐week‐old male C57BL/6 mice into five treatment groups: vehicle (PBS), UMSCs, UMSC exosomes, B2M^‐^UMSCs and B2M^‐^UMSC exosomes. Under general anaesthesia by isoflurane inhalation (2%‐4% isoflurane in oxygen), the left femoral artery was ligated by placing two adjacent sutures around the femoral artery, proximal to the origin of the femoral bifurcation. The mice received a single intramuscular injection of one of the above treatments into the gastrocnemius muscle of the ischaemic hindlimb 24 hours after surgery. Motor function and limb salvage were scored on a scale of 1‐5 (1, poor; 5, strong) as previously described.^26^ At day 28, mice were anesthetized and bodyweight and muscle mass were measured.

### 2.3 Laser Doppler perfusion imaging

We used a laser Doppler imaging device (Moor Instruments) to measure the perfusion at 0, 7, 14, 21 and 28 days in all treatment groups. Perfusion was expressed as the perfusion ratio in the ischaemic leg compared with the contralateral, non‐injured leg.^27^ We focused our measurements on regional perfusion from ankle to toe because the extremities are most affected by ischaemic injury.

### 2.4 Running endurance

The run‐to‐exhaustion performance test was used to assess whether the improvement of perfusion in B2M^‐^UMSCs‐treated mice is associated with enhanced muscle strength and long‐term function. At day 28, mice were exercised following a standard run‐to‐exhaustion protocol as described previously.^27^ Briefly, mice were acclimated to the treadmill (Jiangsu SANS Biological Technology Co. Ltd.) for 1‐2 hours and to the motor sound for 15 minutes before the exercise started. The initial speed was set at 6 m/min and then incrementally increased 2 m every 2 minutes until reaching 18 m/min. Exhaustion was defined as the point where mice spent more than 10 seconds on the shock grid without re‐engaging the treadmill.

### 2.5 Muscle force measurement

Muscle force was measured by grip strength meter as described previously.^28^ The mice were placed on the grip plate. After the animals grasped the grip plate, they were gently pulled back by grasping the tail, causing the animal to loosen the claws. The maximum grip of each mouse was automatically recorded by the instrument. Mouse grip strength was measured daily for 3 consecutive days using a grip strength meter (Ji‐Nan Biotechnology, Shandong, China). Each day, six grip strengths were assessed at 1‐minute intervals, and the average grip strength over 3 days was calculated.

### 2.6 Muscle mass measurement

The mice were killed by CO_2_ inhalation at the end of the experiments, and then gastrocnemius muscles were isolated and weighed. Finally, the gastrocnemius muscle weight relative to bodyweight was calculated as muscle mass/bodyweight ratio.

### 2.7 B2M knockout

To assess the effectiveness of B2M knockout in blunting the immune response, we constructed a lentivirus expressing CRISPR/Cas9 and a CRISPR guide RNA (the synthetic oligos to generate gRNA forward GACCGAGTCACATGGTTCACACGGC; reverse AAACGCCGTGTGAACCATGTGACTC) targeting B2M. The lentivirus was constructed by ligating the sgRNA targeting the B2M sequence to the lenti CRISPR V2 lentiviral vector. The positive recombinant plasmid together with packaging plasmids ΔR8.74, VSV‐G and Rev were cotransfected into 293T cells to generate lentivirus particles.^29^ The packaging plasmids were provided by Dr Yun Zhao, Soochow University. The virus supernatant was collected, concentrated and tittered. UMSCs of passages 2‐3 were subcultured in a 10 cm cell culture dish at a ratio of 1:2 (1 × 10^6^/dish). Viral transfection was carried out at a density of about 50% confluency on the second day. After 48 hours of transfection, the cells were selected by puromycin for another 48 hours to select the cells that were successfully transfected with the lentivirus. Approximately 30% to 40% of UMSCs survived the selection.

### 2.8 Luciferase reporter assays

To determine whether miR‐24 regulates Bim transcription, we constructed a reporter plasmid carrying 3′UTRs of the Bim gene, which was amplified by PCR and inserted into the firefly luciferase reporter psiCHECK^TM‐2^ vector (Promega). The Bim primers used were forward: CCGCTCGAGCTGTGTCGATGTGGACGGAA, reverse: TTGCGGCCGCAGGGCAACGCCATACTCTTC. The miR‐24 overexpression vector LV3‐miR‐24 was constructed by inserting the miR‐24 sequence S: GATCCGTGGCTCAGTTCAGCAGGAACAGTTCAAGAGACTGTTCGCTGAACTGAGCCACTTTTTTG, AS: AATTCAAAAAAGTGGCTCAGTTCAGCGAACAGTCTCTTGAACTGTTCCTGCTGAACTGAGCCACG into the LV3 vector (LV3:pGLV‐H1‐GFP + Puro Vector). HEK293T cells were transfected with 200 ng psiCHECK^TM‐2^ vector‐3′UTR, or a control plasmid expressing luciferase driven Bim 3' UTR mut, and 600 ng LV3‐miR‐24 vector using Lipofectamine 2000 in 24‐well plates for 48 hours. The activity of firefly and Renilla luciferase was analysed by a dual‐luciferase reporter assay kit (Promega) according to the manufacturer's instructions.

### 2.9 Western blot

Protein samples were extracted from UMSCs or exosomes using RIPA buffer (Beyotime Biotechnology) containing a cocktail of protease inhibitors (Beyotime Biotechnology). Equal amounts of protein were separated by SDS‐polyacrylamide gel electrophoresis and then transferred to PVDF membranes. The membranes were blocked with 5% bovine serum albumin (BSA) for 1 hour at room temperature and then incubated with primary antibodies followed by a horseradish peroxidase‐conjugated secondary antibody. The primary antibodies used were B2M (1:5000; Abcam Inc) and Bim (1:1000; Cell Signaling Technology Inc). The proteins were visualized using an ECL chemiluminescence kit (Biological Industries), and the luminescence was detected using a BioRad luminescent imaging system.

### 2.10 Electron microscopy

To reveal the morphology of the exosomes by transmission electron microscopy, exosomes were re‐suspended in PBS, placed on copper grids and incubated for 30 minutes as described previously.^30^ The samples were washed several times with deionized water before incubating with 2% uranyl acetate for 15 minutes. Samples were embedded in a medium containing 0.13% methyl cellulose and 0.4% uranyl acetate for 10 minutes. Each grid was examined in a JEOL JEM 1230 transmission electron microscope, and images were captured using a CCD digital camera.

### 2.11 Loading of miR‐24 mimic into exosomes

miR‐24 mimic was loaded into exosomes by electroporation. The loading of exosomes with the miRNA‐24 mimic or a scrambled mimic was performed based on a previously optimized protocol.^30^, ^31^ Briefly, the exosomes were diluted in the P1 primary cell solution (Lonza) at a final concentration of 1 μg/μL. The human miRNA‐24 mimic (RiboBio) or a scrambled miRNA mimic control (500 pmol) was added to 200 μL of exosome sample containing 1 μg/μL exosomal protein. The mixtures were transferred into cold electroporation cuvettes and electroporated using the 4D‐Nucleofector™ system (Lonza). After that the mixture was treated with one unit of RNase A (Takara) for 30 minutes in order to degrade the excess miRNA mimic. RNase A was then deactivated by adding 2 μL RNase inhibitor (Takara), and the exosomes were re‐isolated using the total exosome isolation kit (Life Technology) according to manufacturer's instructions. The final pellet (exosomes) was re‐suspended in PBS, divided into 100 μL aliquots and stored at −80°C.

### 2.12 miRNA quantification

Total RNA from UMSCs, B2M^‐^UMSCs and their respective exosome preparations were extracted using the TRIzol reagent (Life Technologies). RNA concentrations were measured using a spectrophotometer (NanoDrop). Equal amounts of RNA (1 μg) were reverse‐transcribed using the Revert Aid First Strand cDNA Synthesis kit (Thermo Scientific). The Bulge‐loop^TM^ miRNA qRT‐PCR Primer Sets (one RT primer and a pair of qPCR primers for each set) specific for has‐miR‐24, cel‐miR‐39 and U6 were designed by RiboBio. The expression levels of miRNAs were analysed by qRT‐PCR using Takara SYBR Premix Ex Taq (Tli RNaseH Plus) in a StepOnePlus Real‐Time PCR system (Applied Biosystems). The Ct values of cells were averaged and normalized to the U6 RNA, and the Ct values of exosomes were averaged and normalized to the cel‐miRNA‐39.^32^ All experiments were repeated at least three times. Relative expression was determined by the _ΔΔ_Ct comparative threshold method.

### 2.13 miRNA sequencing and analysis

The procedures for miRNA sequencing have been described in detail previously by our group.^20^ Differentially expressed genes were defined using a FDR (false discovery rate) threshold and log_2_FC (fold change) analysis through EBSeq algorithm. The threshold was defined as FDR < 0.05 and log_2_FC > 1 or <−1. The target gene of miRNA was predicted by TargetScan and Miranda software. The predicted targets of the differentially expressed miRNAs were then analysed by gene ontology (GO) categories and pathways using Fisher's exact test and *χ^2^* test.

### 2.14 miR‐24 inhibitor synthesis and administration

The miR‐24 inhibitor was designed and synthesized as unconjugated and fully phosphorothiolated oligonucleotides by RiboBio. The miR‐24 inhibitor or negative control (scrambled oligonucleotide) at a final concentration of 100 nmol/L was transfected into B2M^‐^UMSCs using Lipofectamine 2000 for 48 hours.

### 2.15 ELISA

Serum TNF‐α level was analysed by ELISA per manufacturer's instructions (TNF‐α ELISA kit: eBioscience Cat# BMS607/3 RRID:AB2575663).

### 2.16 Cytolytic assay

To investigate how T cells react to UMSCs and B2M^‐^UMSCs, we analysed the cytolytic activity of CD8^+^ T cells against UMSCs and B2M^‐^UMSCs at various effector cell/target cell (E/T) ratios. The cytolytic assay was performed as previously described.^6^ The CD8^+^ T cells involved in the cytotoxicity experiments were from healthy volunteers with signed informed consent. Briefly, CD8^+^ T cells were isolated from human peripheral blood mononuclear cells by Ficoll density gradient centrifugation followed by further purification using the CD8^+^ T‐Cell isolation kit (Stem Cell Technologies). The cells were cultured in RPMI 1640 containing 10% FBS supplemented with 50 U/mL human IL‐2 for 48 hours before assay. The cytotoxic activity of CD8^+^ T cells against UMSCs and B2M^‐^UMSCs was determined using the cytotoxicity detection kit (Roche Applied Science). The UMSCs and B2M^‐^UMSCs were re‐suspended at a concentration of 2 × 10^6^/mL in the assay medium, and 100 μL of cell suspension was added to each well of a 96‐well plate. The CD8^+^ T cells in 100 μL of assay medium were then added and mixed with the target cells at various effector/target (E/T) ratios (25:1, 12.5:1, 6.25:1 and 3.125:1). After co‐culture for 3 hours, the 96‐well plates were centrifuged at 250 *g* for 10 minutes, and supernatant was transferred onto a new 96‐well plate, mixed with 100 μL reaction mixture and incubated for 30 minutes at room temperature. The reaction was terminated, and the absorbance of the samples was measured at 490 nm.

### 2.17 Immunofluorescence staining of CD8 and Complement C3

To assess the effectiveness of B2M knockout in blunting the immune response, we analysed CD8 and complement C3 levels in muscle sections by immunofluorescence assay. Gastrocnemius muscles were isolated, frozen in isopentane cooled in liquid nitrogen and sectioned on a microtome cryostat. Frozen sections (6 μm) were incubated with rabbit anti‐CD8 antibody (1:100; Bioss Inc) or rabbit anti‐complement C3 antibody (1:100; Boster Biological Technology. Catalog #A00168‐1) overnight at 4°C. After washing, the sections were incubated with goat anti‐rabbit IgG (ab150077 Alexa Fluor^®^ 488, Abcam) at 2 µg/mL for 1 hour. The sections were finally incubated with DAPI to stain nuclei.

### 2.18 Immunofluorescence staining of HLA‐I

In order to detect the expression of HLA‐I, UMSCs and B2M^‐^UMSCs were fixed in 4% paraformaldehyde for 30 minutes at room temperature and then blocked with 5% BSA in PBS for 1 hour at room temperature. The cells were incubated with mouse anti‐human HLA class I mAb (DAKO) at 1:100 dilution in 5% BSA overnight at 4℃. After washing, the cells were incubated with the secondary antibody goat antimouse IgG (ab150113 Alexa Fluor^®^ 488, Abcam) at 2 µg/mL for 1 hours at room temperature. Nuclei were stained with DAPI.

### 2.19 Atomic force microscopy (AFM)

Atomic Force Microscopy was employed to determine the proliferation of UMSCs and B2M^‐^UMSCs. It has been shown that cell morphology determines its proliferation and adhesion.^33^ Prockop et al^34^, ^35^ showed that mesenchymal stem cells can be divided into two types: fast self‐renewal, small, round or spindle‐like cells, and slow growth, large, cubic or flat cells. AFM measurements were performed using a NanoWizard atomic force microscope (JPK Instruments) mounted on the modified stage of an inverted optical microscope (Axiovert 200, Carl Zeiss, MicroImaging), which was used to select a desired cell and to position the AFM tip. UMSCs and B2M^‐^UMSCs were fixed in 2.5% glutaraldehyde for 5 minutes at room temperature and imaged with 1024 × 1024 pixels at line rates of 0.3‐0.5 Hz.

### 2.20 Flow cytometry

Both regular flow cytometry and imaging flow cytometry were employed to analyse the expression of HLA‐I. Interferon gamma (IFN‐γ) is a typical inflammatory factor that is known to induce the expression of HLA‐I.^36^ UMSCs and B2M^‐^UMSCs were treated with IFN‐γ (25 ng/mL) for 48 hours, then digested with 0.25% trypsin and suspended with PBS (10^6^ cells/mL). The cells were incubated with the anti‐HLA‐I antibody for 30 minutes, followed by AF488 labelled secondary antibody. The expression of HLA‐I was analysed by regular flow cytometry. The data revealed that HLA‐I was expressed on UMSCs but was not detectable on B2M^‐^UMSCs.

To further confirm that knockout of B2M blocks the expression of HLA‐I, we performed imaging flow cytometry. Data were acquired on an imaging flow cytometer (ImageStreamX; Amnis/EMD Millipore), including a bright field image. AF488 was excited by a 488‐nm laser with a 100‐mW output, DAPI by a 405‐nm laser with a 10‐mW output and AF546 by a 546‐nm laser with a 50‐mW output. The selected laser outputs prevented saturation of pixels in the relevant detection channels as monitored by the corresponding Raw Max Pixel features during acquisition. For each sample, bright field, HLA‐Ⅰ‐AF488 with total CD90‐AF546, and DAPI images were simultaneously collected for 20 000 events.

### 2.21 Statistical analysis

The data are expressed as the mean ± SEM. Multiple comparisons were analysed by ANOVA with post hoc analysis by the Newman‐Keuls test. Two‐tailed t tests were used to determine the significance of differences between two groups. *P* < .05 was considered statistically significant.

## 3 RESULTS

### 3.1 Generation and characterization of B2M^‐^UMSCs

**Figure 1:**
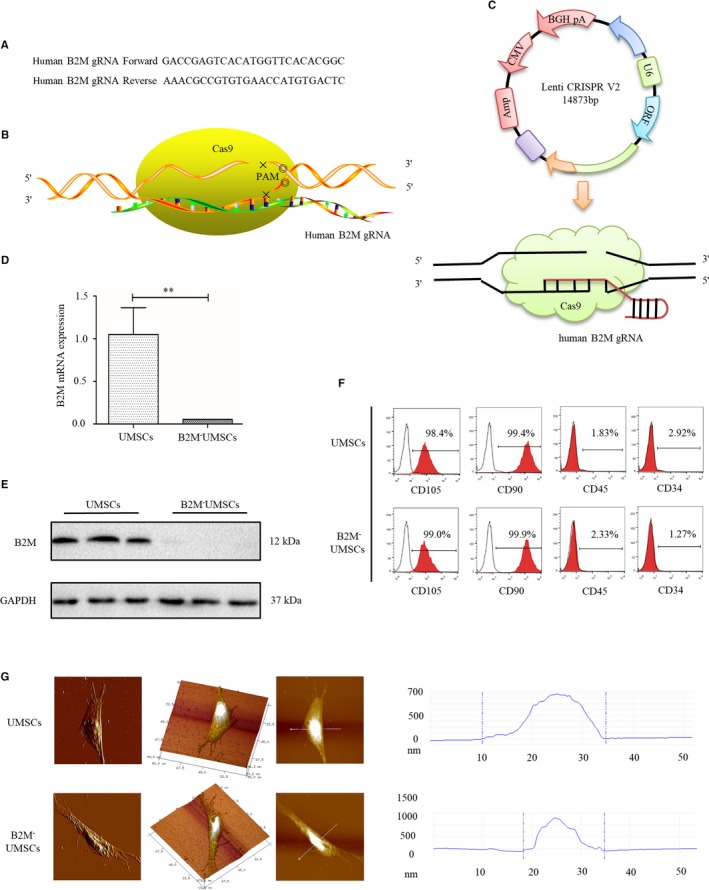
Generation and characterization of B2M^‐^UMSCs. A, Human gRNA (guide RNA) sequence targeting B2M. B, Diagram of B2M knockout by CRISPR/Cas9. C, Lenti CRISPR V2 lentiviral plasmid map. (D,E) RT‐PCR and Western blots of B2M expression in UMSCs or with B2M knockout. **P < .01. F, Flow cytometry analysis of the expression of MSC markers. G, Atomic force microscopy to determine the proliferation of UMSCs and B2M^‐^UMSCs

**Figure 2:**
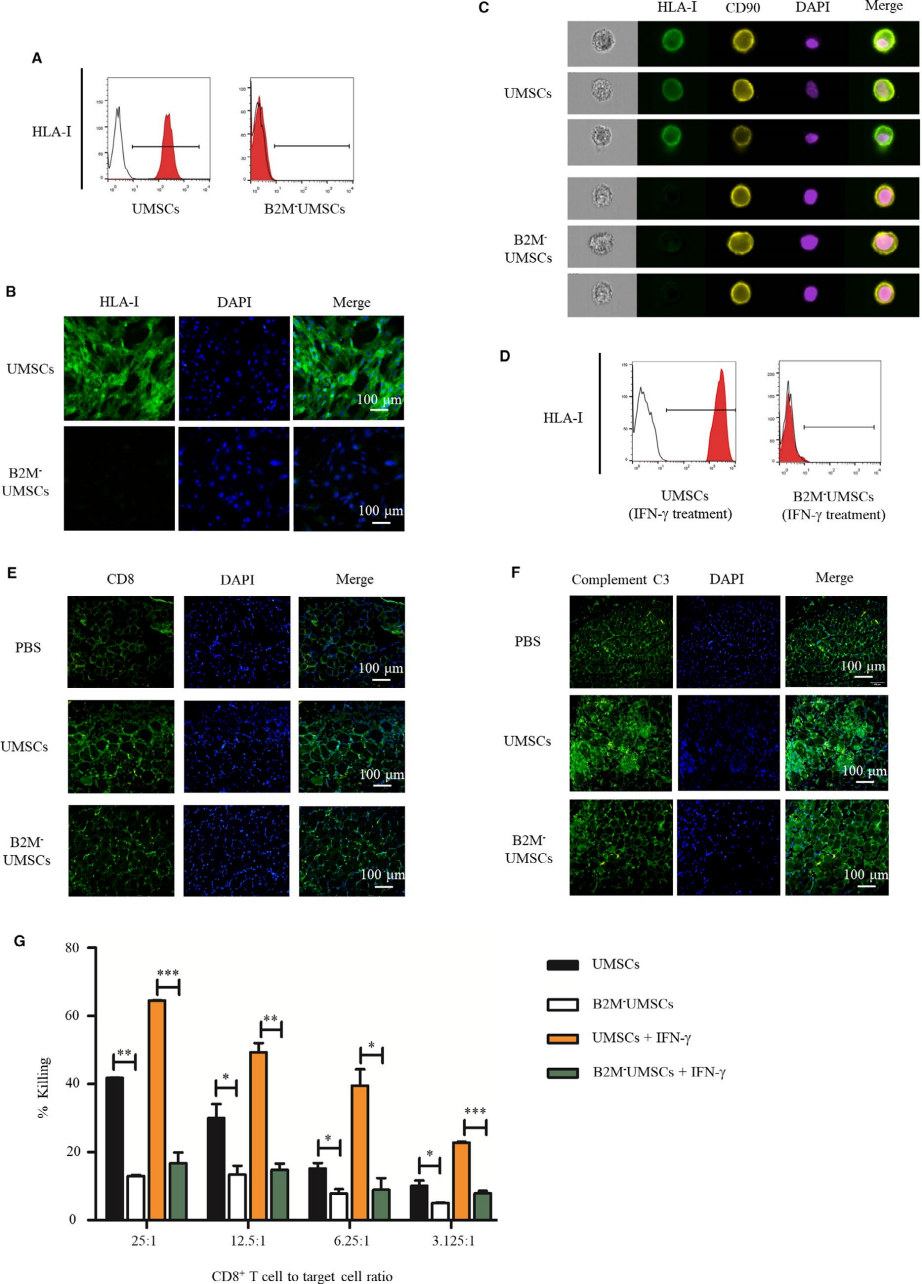
Knockout of B2M in UMSCs inhibits immune rejection. A, Flow cytometry to analyse surface HLA‐I protein expression in UMSCs and B2M^‐^UMSCs. (B,C) Immunofluorescence staining and imaging flow cytometry to analyse the expression of positive HLA‐I on the surface of UMSCs and B2M^‐^UMSCs (scale bar, 100 μm). D, Flow cytometric analysis of HLA‐I expression in the presence of IFN‐γ. E, The number of CD8^+^ T cells in the gastrocnemius muscle sections was analysed by immunofluorescence (green) (N = 3/group; scale bar, 100 μm). F, The detection of complement C3 levels (green) in muscle sections by immunofluorescence assay (N = 3/group; scale bar, 100 μm). G, Cytolytic activity of CD8^+^ T cells against UMSCs and B2M^‐^UMSCs at various E/T ratios with or without IFN‐γ. *P < .05, **P < .01, ***P < .001

A lentivirus targeting B2M was constructed using CRISPR/Cas9 technology (Figure 1A,B). B2M ablation was achieved by transfecting lentivirus particles into UMSCs (Figure 1C). The ablation of B2M was confirmed by PCR (Figure 1D) and Western blot analysis (Figure 1E). B2M knockout did not alter the expression of MSC markers such as CD90 and CD105 (Figure 1F) or the typical spindle‐shaped morphology, but the average height of nucleus area of B2M^‐^UMSCs was increased compared to UMSCs as determined by atomic force microscopy, suggesting increased cell proliferation when B2M was deleted (Figure 1G). The data from flow cytometry (Figure 2A), immunofluorescence (Figure 2B) and imaging flow cytometry (Figure 2C) confirmed that the expression of HLA‐I was blocked by B2M deletion. HLA‐I expression was not increased in B2M^‐^UMSCs even after IFN‐γ stimulation (Figure 2D), indicating that the disruption of HLA‐I by B2M deletion was successful.

### 3.2 B2M deletion in UMSCs inhibits immune rejection

In order to determine whether UMSC injection induces immune rejection in mice with a normal immune system, we injected UMSCs into the ischaemic hindlimb of wild‐type C57BL/6 mice and the data revealed that UMSCs induced an immune response as evidenced by increased TNF‐α level after 3 days (UMSCs = 974 ± 25 pg/mL vs PBS = 728 ± 39 pg/mL, *P* < .05).

To assess the effectiveness of B2M knockout in blunting the immune response, we analysed the recruitment of CD8^+^ T cells in muscle sections by immunofluorescence assay. UMSCs increased the recruitment of CD8^+^ T cells in ischaemic muscle compared to PBS treatment, but the number of CD8^+^ T cells was reduced in B2M^‐^UMSCs‐treated group compared to UMSC treatment (Figure 2E). Moreover, when B2M^‐^UMSCs were injected into the ischaemic leg of immunocompetent mice, these cells did not induce an immune response as determined by the TNF‐α level (B2M^‐^UMSCs = 732 ± 26 pg/mL vs PBS = 728 ± 39 pg/mL, P = NS) and complement C3 levels (Figure 2F).

To further investigate how T cells react to UMSCs and B2M^‐^UMSCs, we analysed the cytolytic activity of CD8^+^ T cells against UMSCs and B2M^‐^UMSCs at various effector cell/target cell (E/T) ratios (Figure 2G). The results showed that CD8^+^ T cells caused significant killing of UMSCs at all E/T ratios, with the most vigorous killing occurring at the highest concentration of CD8^+^ T cells (25:1). By contrast, no CD8^+^ T cell‐mediated killing of B2M^‐^UMSCs was observed even at the highest E/T ratio (Figure 2G), indicating the non‐immune nature of B2M^‐^UMSCs. CD8^+^ T cell‐mediated cytotoxicity was barely detectable in B2M^‐^UMSCs even when the cells were treated with IFN‐γ (Figure 2G). Collectively, these data demonstrated that B2M^‐^UMSCs do not induce CD8^+^ T cell‐mediated immune response.

### 3.3 Knockout of B2M increased the retention rate of UMSCs in vivo

**Figure 3:**
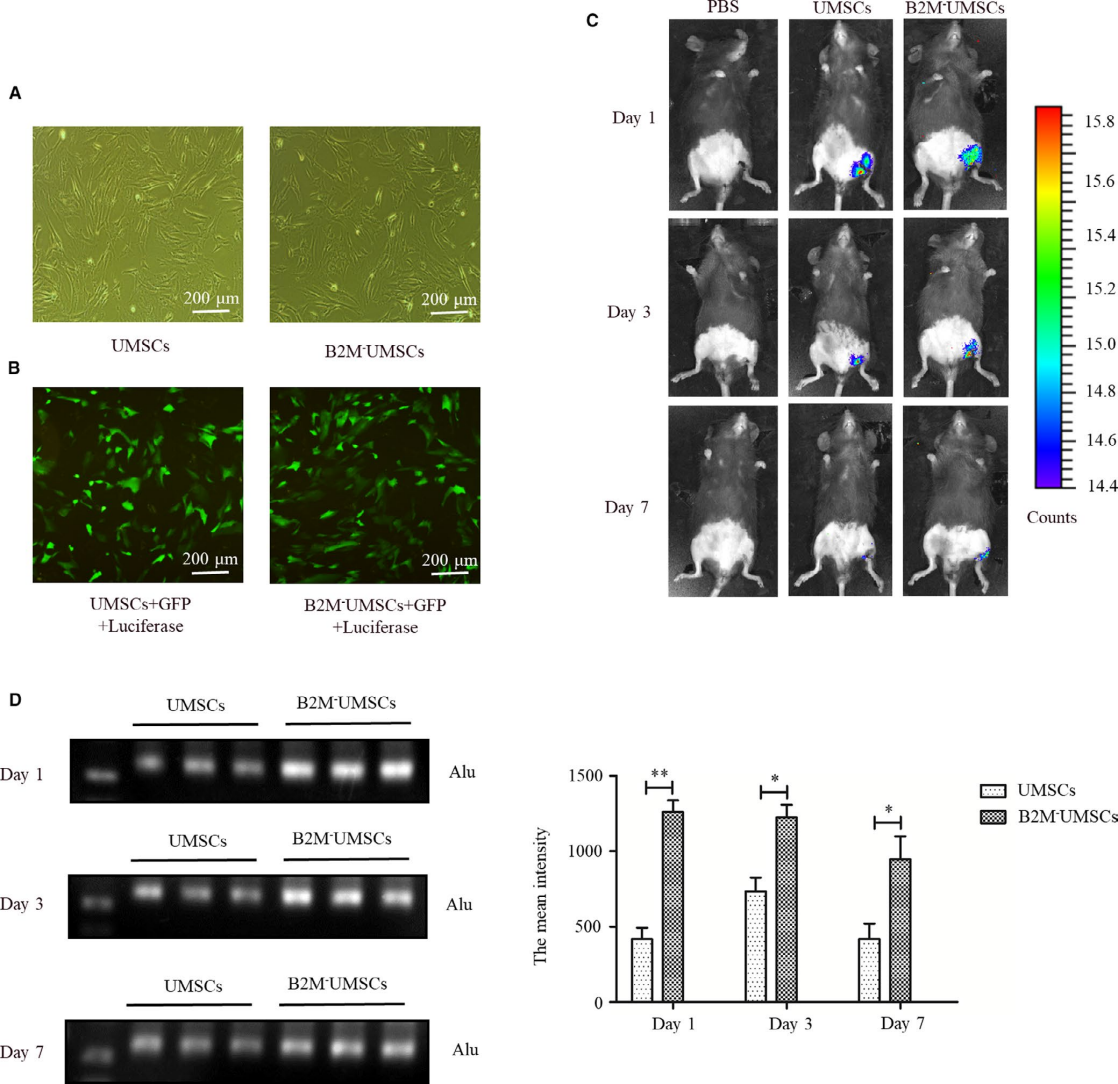
The retention rate of UMSCs and B2M^‐^UMSCs in vivo. A, Transfection of a luciferase reporter into both UMSCs and B2M^‐^UMSCs. B, Fluorescence microscopic images of UMSCs and B2M^‐^UMSCs transfected with lentiviral vector carrying GFP and a luciferase reporter gene (scale bar, 200 μm). C, The retention rate of UMSCs and B2M^‐^UMSCs in vivo was analysed by whole‐body imaging. D, PCR analysis of the expression of Alu in mouse gastrocnemius after transplantation. *P < .05, **P < .01

To access the retention of the transplanted stem cells, we transfected a luciferase reporter into both UMSCs and B2M^‐^UMSCs in order to track their location in vivo and found that the transfection efficiency was similar under light (Figure 3A) and fluorescence microscopy (Figure 3B). These cells were injected into leg muscles, and whole‐body imaging showed that they were only found in the injection site, and the luminescence intensity was stronger in B2M^‐^UMSC group (Figure 3C). Moreover, PCR was performed (with equal quantity template) to analyse the expression of Alu, a repeated sequence specially expressed in human but not in mouse. The results showed that there were more B2M^‐^UMSCs retained in the ischaemic hindlimb than UMSCs 1, 3 and 7 days after transplantation (Figure 3D).

### 3.4 Paracrine effects of B2M^‐^UMSCs

**Figure 4:**
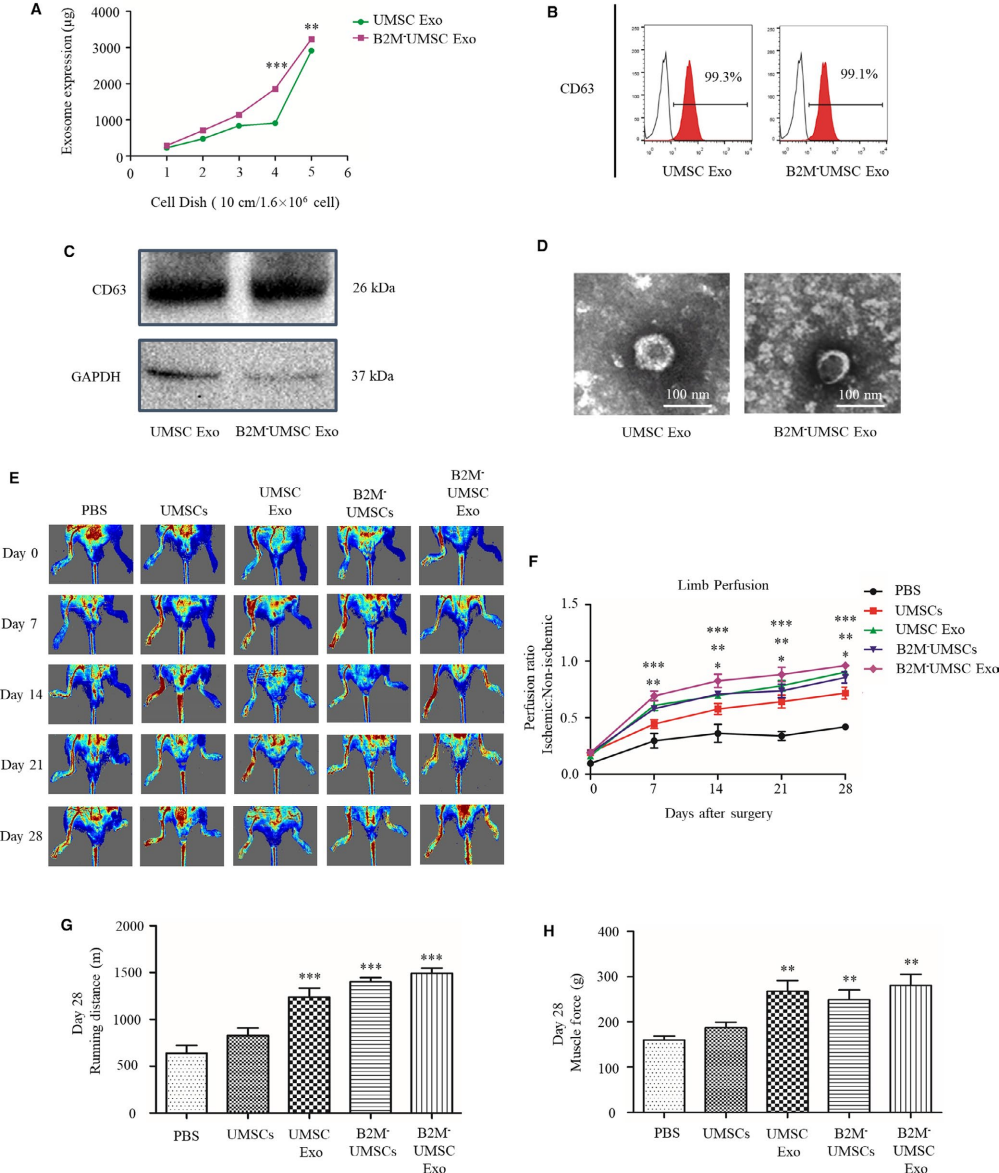
B2M^‐^UMSCs and exosomes enhance ischaemic hindlimb repair. A, The protein concentration of UMSCs and B2M^‐^UMSCs‐derived exosomes was determined by BCA assay. B, Expression of the exosome marker CD63 was analysed by flow cytometry. C, Western blots of CD63 protein in exosomes from UMSCs and B2M^‐^UMSCs. D, Morphology of exosomes from UMSCs and B2M^‐^UMSCs revealed by electron microscopy (scale bars, 100 nm). E, Laser Doppler perfusion imaging of limbs from mice in different treatment groups after ischaemic surgery. F, Blood flow recovery after ischaemic surgery. G, Running distance. H, Muscle force. N = 5/group. *P < .05, **P < .01, ***P < .001

To determine whether knockout of B2M alters the purification yield and properties of exosomes, the protein concentration of UMSCs and B2M^‐^UMSCs‐derived exosomes was determined by BCA assay. Data showed that a good correlation between the number of cells and the protein amount of purified exosomes and the protein concentrations of exosomes secreted by B2M^‐^UMSCs were higher than that of UMSCs (Figure 4A). Meanwhile, we analysed the expression level of the exosome marker CD63 by flow cytometry and Western blot, which showed similar CD63 expression between B2M^‐^UMSCs and UMSCs (Figure 4B,C). Electron microscopic examination of the morphology of exosomes derived from B2M^‐^UMSCs and UMSCs showed no difference (Figure 4D).

To determine whether B2M^‐^UMSCs enhance the reparative effect through exosomes, we assessed the efficacy of B2M^‐^UMSCs and their exosomes in restoring blood flow in the ischaemic mouse hindlimb. Sex‐ and age‐matched mice received a single intramuscular injection of one of the following treatment after undergoing the same surgical procedure: (a) vehicle (PBS); (b) UMSCs (4.5 × 10^5^ cells/mouse); (c) UMSC exosomes (100 µg); (d) B2M^‐^UMSCs (4.5 × 10^5^ cells/mouse); and (e) B2M^‐^UMSC exosomes (100 µg). Laser Doppler analysis showed significantly enhanced perfusion at days 7, 14, 21 and 28 in B2M^‐^UMSCs and B2M^‐^UMSC‐exosome treated mice (Figure 4E,F). Treadmill testing showed improved running endurance in mice injected with B2M^‐^UMSCs and their exosomes. The motor and salvage scores were higher in B2M^‐^UMSCs and exosomes treatment groups (Figure S1A,B) (*P* < .05 for B2M^‐^UMSCs versus UMSCs；*P* < .001 for B2M^‐^UMSC Exo vs PBS). At day 28, the running distance was 654.9 ± 48.90 m for PBS, 827 ± 74.92 m for UMSCs, 1211.13 ± 216.10 m for UMSC exosomes, 1402 ± 71.05 m for B2M^‐^UMSCs and 1525 ± 63.92 m for B2M^‐^UMSC exosomes (Figure 4G) (*P* < .001 for PBS vs B2M^‐^UMSCs; PBS vs UMSC Exo; PBS vs B2M^‐^UMSC Exo; UMSCs vs B2M^‐^UMSCs; UMSCs vs UMSC Exo; UMSCs vs B2M^‐^UMSC Exo); the running time was 41.33 ± 2.96 minutes for PBS, 52.67 ± 4.80 minutes for UMSCs, 76.67 ± 13.53 minutes for UMSC exosomes, 88.67 ± 4.41 minutes for B2M^‐^UMSCs, and 96 ± 4.04 minutes for B2M^‐^UMSC exosomes (Figure S1C) (*P* < .01 for PBS vs UMSCs; *P* < .001 for PBS vs B2M^‐^UMSCs; PBS vs UMSC Exo; PBS vs B2M^‐^UMSC Exo; UMSCs vs B2M^‐^UMSCs; UMSCs vs UMSC Exo; UMSCs vs B2M^‐^UMSC Exo) (*P* < .001). The data showed that the motor and salvage scores, running distance and running time were significantly improved by B2M^‐^UMSCs and their exosomes.

Furthermore, muscle force (Figure 4H) and the muscle mass/bodyweight ratio (Figure S1D) were also improved in the groups treated with B2M^‐^UMSCs and their exosomes. Taken together, these data showed that treatment with B2M^‐^UMSCs and their exosomes are superior to PBS in improving functional recovery in the ischaemic mouse hindlimb.

### 3.5 The therapeutic effects of B2M^‐^UMSCs and exosomes are mediated by miR‐24

**Figure 5:**
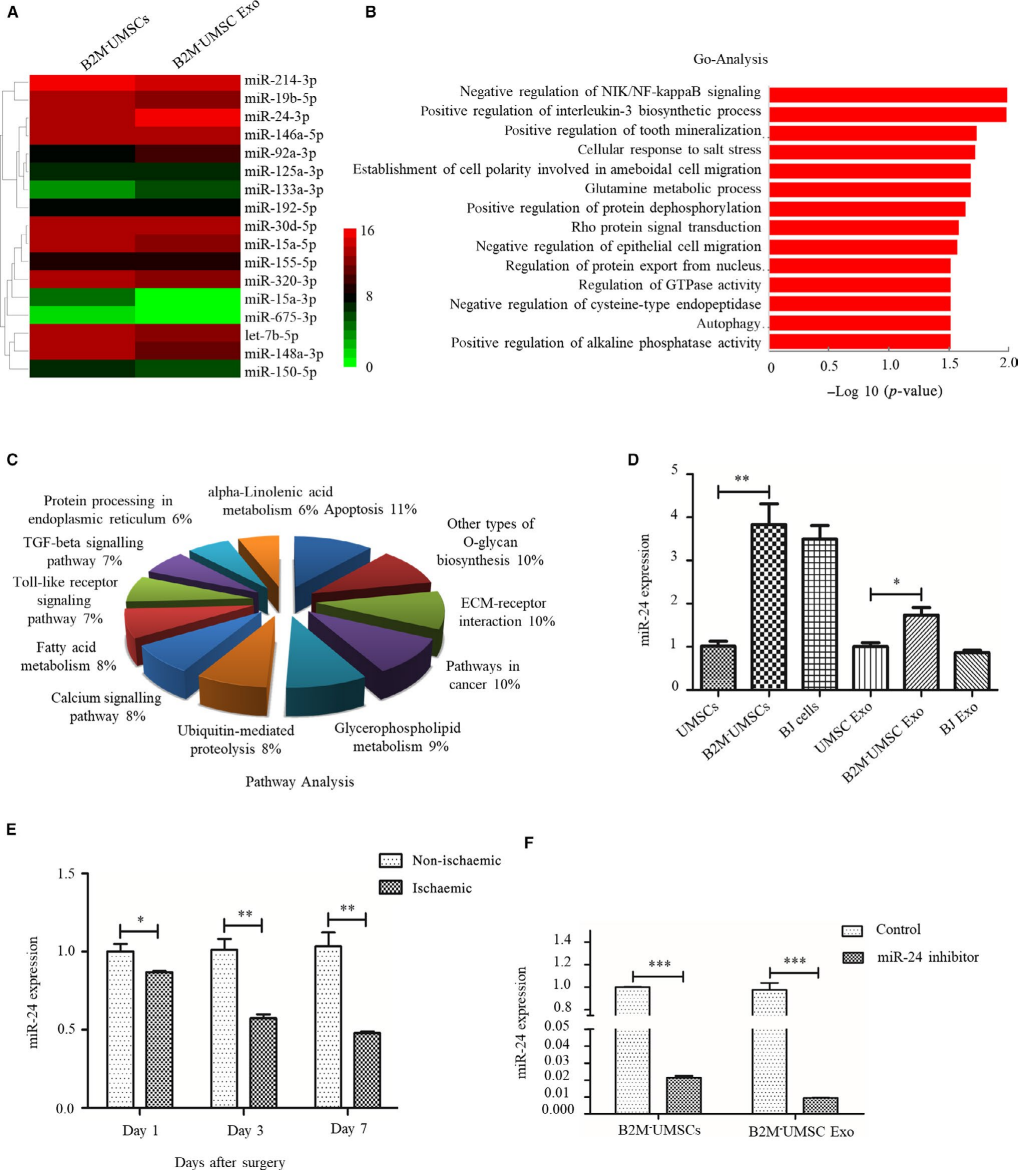
Analysis of the expression of miR‐24 and associated pathway. A, Heatmap of miRNA sequencing data from B2M^‐^UMSCs and B2M^‐^UMSC exosomes (green, downregulated; red, upregulated). B, Gene ontology (GO) in B2M^‐^UMSC exosomes compared with B2M^‐^UMSCs. C, Analysis of pathways involving miRNAs. D, RT‐qPCR analysis of miR‐24 expression in cells and their derived exosomes. *P < .05, **P < .01. E, Real‐time PCR assessment of miR‐24 expression in ischaemic gastrocnemius at 1, 3 and 7 days. N = 3; *P < .05, **P < .01. F, Real‐time PCR analysis of miR‐24 expression in B2M^‐^UMSCs and B2M^‐^UMSC exosomes after transfection of the miR‐24 inhibitor compared to control. N = 3/group; ***P < .001

**Figure 6:**
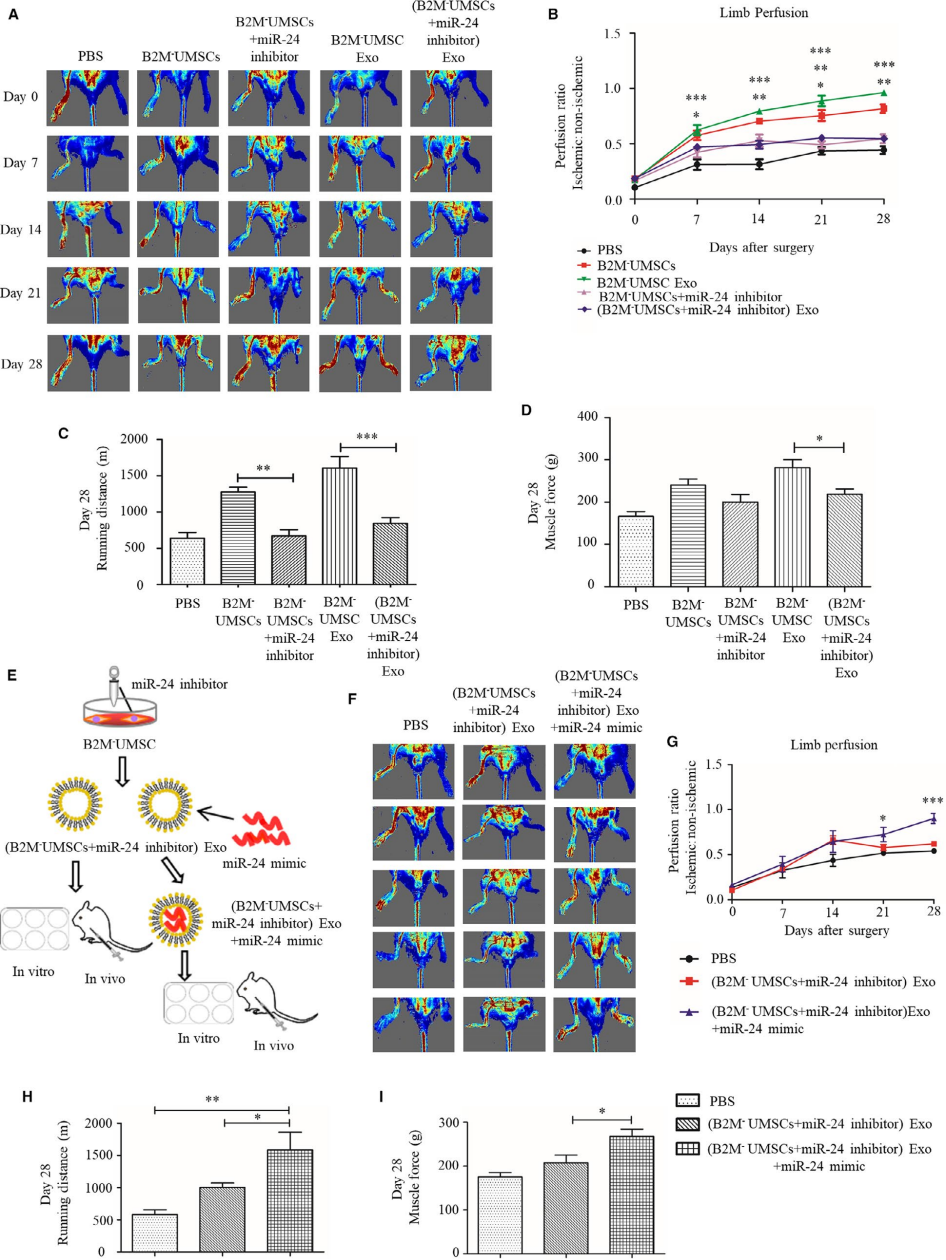
miR‐24 is necessary for revascularization in mouse hindlimb. A, Laser Doppler perfusion images of blood flow from mice with different treatment groups after ischaemic surgery. B, Blood flow recovery. C, The effect of miR‐24 inhibitor on running distance. D, The effect of miR‐24 inhibitor on muscle force. E, Flowchart of miR‐24 mimics loading into B2M^‐^UMSC exosomes. F, Laser Doppler perfusion images. G, Limb perfusion. H, The effect of miR‐24 inhibitor and mimic on running distance. I, The effect of miR‐24 inhibitor and mimic on muscle force. N = 6/group. *P < .05, **P < .01, ***P < .001

To delineate the molecular mechanisms underlying the beneficial effects of B2M^‐^UMSCs and exosomes, we focused on miRNA. We performed hierarchical cluster analysis of miRNA expression in order to further characterize the differential expression of miRNA between B2M^‐^UMSCs and B2M^‐^UMSC Exo (Figure 5A). The predicted targets of the differentially expressed miRNAs were then analysed in terms of their gene ontology (GO) categories (Figure 5B) and pathways (Figure 5C) using Fisher's exact test and χ^2^ test. Our results showed that B2M^‐^UMSC exosomes and B2M^‐^UMSCs have a similar miRNA sequencing profile in general, and the expression of miR‐24 was particularly high, which was verified by RT‐qPCR (Figure 5D). Pathway analysis indicated that apoptosis may play an important role (Figure 5C). We focused on miR‐24 because it prevents apoptosis.^14^ We also found that the expression of miR‐24 became downregulated on the first day of left‐limb ischaemia in mice, and the downregulation even more significant on the 3rd and 7th day (Figure 5E) (*P* < .01). To investigate the role of miR‐24 in tissue repair, we added a miR‐24 inhibitor to B2M^‐^UMSCs and found that it significantly reduced the expression of miR‐24 in B2M^‐^UMSCs and the exosomes compared to control (Figure 5F) (*P* < .001). Importantly, the miR‐24 inhibitor blocked the improvement in blood perfusion, treadmill performance, limb salvage score, muscle force and muscle mass/bodyweight ratio afforded by B2M^‐^UMSCs and the exosomes (Figure 6A‐D, Figure S2A‐D).

To confirm the role of miR‐24 in tissue repair, we took a gain‐of‐function approach to overexpress miR‐24 mimic in exosomes by electroporation (Figure 6E). The miR‐24 inhibitor blocked the improvement of blood perfusion, treadmill performance, limb salvage score, muscle force and muscle mass/bodyweight ratio afforded by exosomes secreted by B2M^‐^UMSCs. In contrast, the miR‐24 mimic restored the beneficial effects of these exosomes in the presence of the miR‐24 inhibitor (Figure 6F‐I, Figure S3A‐D).

### 3.6 Bim is a downstream target of miR‐24

**Figure 7:**
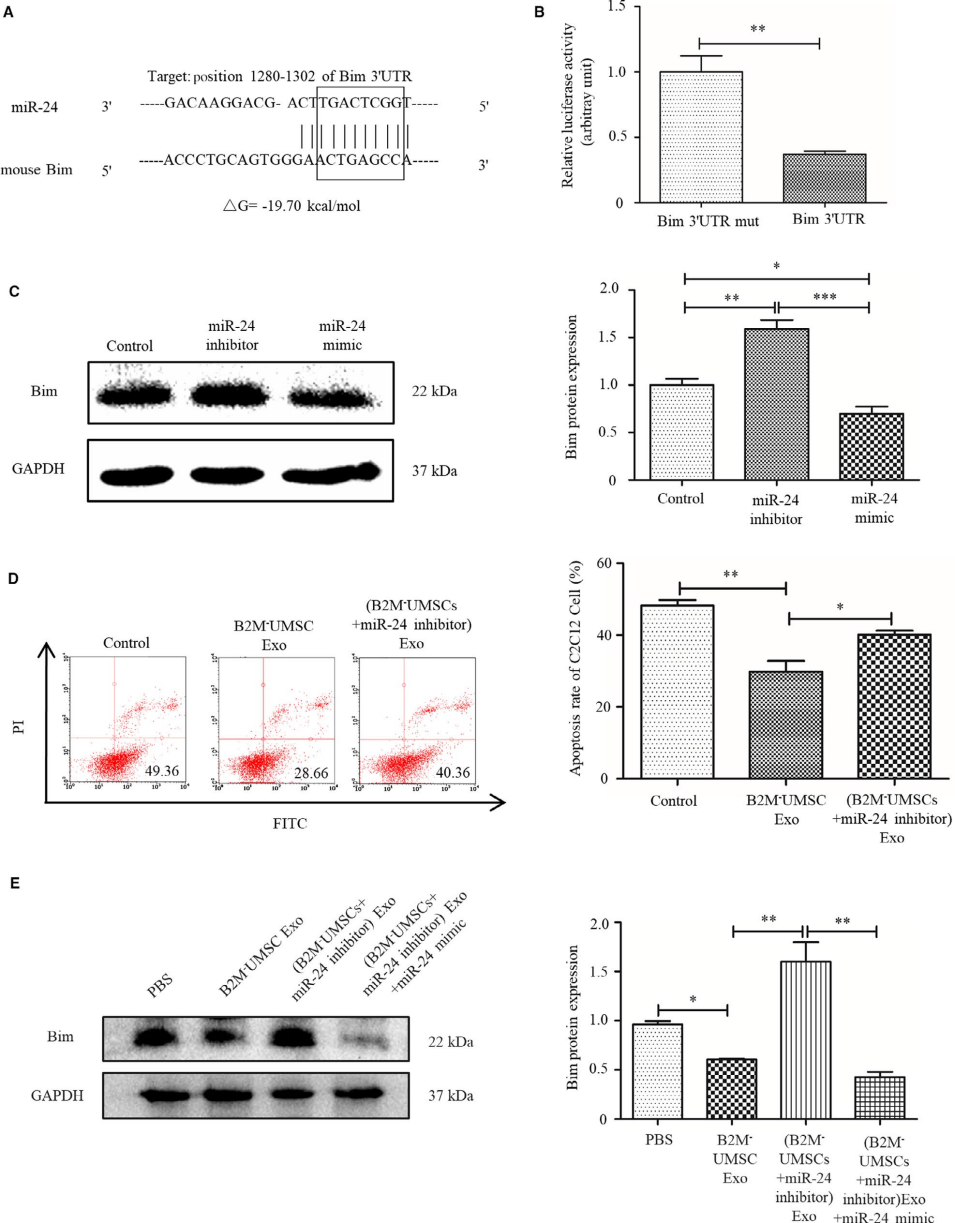
Bim is a target gene of miR‐24. A, TargetScan and RNA22 prediction of conserved miR‐24 binding sites in the Bim 3′ UTR of mouse. B, Luciferase assays of HEK293T cells cotransfected with psiCHECK^TM‐2^ vector‐3′ UTR or a mutated Bim 3’UTR and LV3‐miR‐24 vector for 48 h. **P < .01. C, Western blot analysis of the expression of Bim in C2C12 cells transfected with a miR‐24 inhibitor or miR‐24 mimic. *P < .05, **P < .01, ***P < .001. D, Flow cytometry analysis of hypoxia‐induced apoptosis rate in C2C12 cells transfected with B2M^‐^UMSC Exo or (B2M^‐^UMSCs + miR‐24 inhibitor) Exo. *P < .05, **P < .01. E, Western blots of Bim expression in the ischaemic gastrocnemius muscles with different exosome treatment 3 d. *P < .05, **P < .01

To investigate the mechanism by which miR‐24 enhances tissue repair, we used the bioinformatics tool TargetScan to identify its putative targets. One potential target was Bim, which is a key gene in apoptosis.^21^, ^30^, ^31^, ^37^ We also used the bioinformatics tool RNA22 to confirm this potential target, and the analysis revealed that Bim ΔG = –19.70 kcal/mol (Figure 7A).

To determine whether miR‐24 regulates Bim transcription, we transfected 293T cells with a plasmid expressing luciferase driven by the 3’untranslated region (UTR) of Bim, or a control plasmid expressing luciferase driven Bim 3' UTR mut. Luciferase assays revealed that Bim transcriptional activity decreased significantly in the presence of the miR‐24 mimic (Figure 7B) (*P* < .01). Moreover, we added the miR‐24 inhibitor or mimic to a C2C12 myoblast cell line. Western blot analysis revealed that the expression of Bim was increased in the presence of the inhibitor (*P* < .01) but decreased in the presence of the mimic (*P* < .05) (Figure 7C). The inhibitor blocked the anti‐apoptotic effect of B2M^‐^UMSC exosomes (*P* < .05) (Figure 7D). Western blot analysis revealed that the expression of Bim in the ischaemic gastrocnemius muscles was increased in the presence of the miR‐24 inhibitor (*P* < .01) but decreased in the presence of the miR‐24 mimic (*P* < .05) (Figure 7E). These data confirmed that Bim, a pro‐apoptotic gene, is a direct target of miR‐24. Our data indicate that miR‐24 promotes cell survival by suppressing Bim, and the miR‐24/Bim pathway is essential in mediating the beneficial effect of the exosomes.

## 4 DISCUSSION

Our study demonstrates that deletion of HLA light chain B2M can prevent alloUMSC‐induced immune rejection. Importantly, we showed that B2M^‐^UMSCs were more effective in restoring blood flow and functional recovery in the ischaemic mouse hindlimb than UMSCs. We further showed that the beneficial effects of B2M^‐^UMSCs in promoting survival and repair were mediated by exosomes that release the miR‐24 targeting apoptotic gene Bim. Moreover, we demonstrate that B2M^‐^UMSCs‐derived exosomes were more effective than UMSC‐derived exosomes in restoring ischaemic hindlimb perfusion.

Previous reports showed that MSCs can be rejected by HLA‐mismatched recipients.^38^ However, others showed that alloUMSCs do not induce immune rejection^39^ using immunodeficient animal models.^26^, ^27^ In the present study, we demonstrate that human UMSCs trigger immune rejection in wild‐type C57BL mice with normal immune system. Our findings are more clinically relevant because most patients who require stem cell transplantation are immunocompetent. The generation of HLA deficient MSCs provides a cell source that can be used ‘off the shelf’ in the clinic without the need for HLA matching.

MSCs express only the HLA‐I but not the HLA‐II antigen and disruption of the B2M gene results in a complete loss of surface HLA‐I expression^6^ and diminished immunogenicity.^6^ Therefore we deleted B2M in UMSCs using CRISPR/Cas9 technology and showed that B2M^‐^UMSCs do not trigger immune response.

The therapeutic effects of MSCs are mediated by paracrine factors which are often transported by exosomes.^15^ Indeed, our data showed that exosomes can improve blood perfusion and motor function of ischaemic mouse hindlimb. These findings are significant because exosomes are not only more convenient to use but are also safer than cells, which are associated with immune rejection and possible formation of teratoma in vivo.^40^ The bi‐lipid layer of exosomes functions as a natural shield for functional molecules within the vesicle.^41^ As we have suggested before, exosomes are influenced by both dominant and recessive imprinting of their parent cells.^42^ It was shown that human CD34^+^ stem cell‐derived exosomes improved ischaemic limb perfusion, capillary density and motor function. These exosomes contain miR‐126‐3p, which enhances angiogenesis by targeting *SPRED1.* Interestingly, the exosomes were more efficiently uptaken by endothelial cells compared to smooth muscle cells and fibroblasts.^26^ These results suggest that the final destination of exosomes may be pre‐determined by their parent cells, which further confirmed the importance of dominant imprinting. Along this line, we showed that exosomes derived from B2M^‐^UMSCs are more efficient than those derived from UMSCs.

Moreover, the content and function of exosomes can be modified by external stimuli. For example, although normal dendritic cells contain HLA class I, class II and T‐cell co‐stimulatory molecules, the exosomes derived from these cells were not able to kill tumours. However, when exosomes from dendritic cells that were pulsed with tumour peptides were injected, the exosomes were able to function as vaccine to prime cytotoxic T cells to eradicate established tumours in mice.^43^ Therefore, we should embrace the opportunities to develop more effective exosome‐based therapies through specific treatment of the parent cells.

It has been shown that miR‐24 mimic can improve myocardial function by inhibiting apoptosis when injected directly into the myocardium in a mouse myocardial infarction model.^21^ While these findings are interesting, the direct injection approach of injecting miR‐24 mimic and transfection reagent may not be feasible for treating patients. To solve this problem, we loaded the exosome with miR‐24 mimic in vitro by electroporation and then injected the exosomes into the muscle. Our data revealed that the exosomes improved blood perfusion and function of mouse hindlimb. We further showed that the beneficial effect was mediated by miR‐24/Bim pathway. Our findings have clinical implications because the content of the exomes can be modified in vitro and then injected intravenously. As mentioned above, exosomes can find their target cells in vivo and can be internalized by the cells without the need of any transfection reagents. Therefore, exosome‐based therapies are promising in developing cell free treatment for tissue injury.

Increased peripheral blood cytokine level is a hallmark of ischaemic injuries, and some cytokines can inhibit stem cell function.^44^ Circulating B2M has been considered a pro‐aging factor that impairs neurogenesis.^45^ Therefore, our strategy to knock out B2M not only improves stem cell function but also may prevent UMSC senescence. Consistent with this notion, we found that the retention rate of UMSCs was increased in vivo when B2M was deleted and that B2M deletion also increased the yield of exosomes secreted by UMSCs. This result suggested that B2M might inhibit cell proliferation. Prockop et al^34^, ^35^ showed that the nucleus of mesenchymal stem cells with strong proliferation ability was very large, and the average height of nucleus was relatively high. Indeed, we showed that the average height of nucleus area of B2M^‐^UMSCs was increased compared to UMSCs as determined by atomic force microscopy, suggesting increased cell proliferation when B2M was deleted (Figure 1G). Consistent with these findings, it was shown that B2M is involved in the pathogenesis of osteoarthritis by inhibiting chondrocyte proliferation.^46^

In summary, our study demonstrates that deletion of B2M in alloUMSCs can prevent immune rejection of the transplanted cells. The beneficial effects were mediated by exosomes/miR‐24/Bim pathway. We also showed that miR‐24 mimic can be loaded into exosomes in vitro and then injected into injured muscles to improve muscle blood perfusion and motor function. Our study shed new light into the mechanisms of stem cell‐based therapy and may pave the way to the development of new strategies to treat ischaemic tissue injury.

## CONFLICT OF INTEREST

The authors confirm that there are no conflicts of interest.

## AUTHOR CONTRIBUTIONS

YL conceived, designed the study, analysed data, and wrote the manuscript. YZ, YW, LS, YZ, WX, BY, FL performed experiments and collected data. XP, CL, BL and XY interpreted data and revised the manuscript. All authors read and approved the final manuscript.

## Data Availability

All data used or analysed during this study are included in this published article.
