# Supplementary material for: Knockout of beta‐2 microglobulin reduces stem cell‐induced immune rejection and enhances ischaemic hindlimb repair via exosome/miR‐24/Bim pathway
Source: J Cell Mol Med. 2019 Nov 15;24(1):695–710. doi: 10.1111/jcmm.14778 (PMC6933348; doi:10.1111/jcmm.14778)
Supplement: Supplementary file 1 [file JCMM-24-695-s001.doc]

**Supplemental File**

Knockout of beta-2 microglobulin reduces stem cell-induced immune rejection and enhances ischemic hindlimb repair via exosome/miR-24/Bim pathway

Yuqing Zhang^1^∣Yanli Wang^1^∣Lianbo Shao^1^∣Xiangbin Pan^2^∣ Chun Liang^3^∣Bin Liu^4^∣Yu Zhang^1^∣Wenping Xie^1^∣Bing Yan^1^∣Feng Liu^1^∣Xi-yong Yu^5^∣Yangxin Li^1^

**Supplemental Figure 1**

**
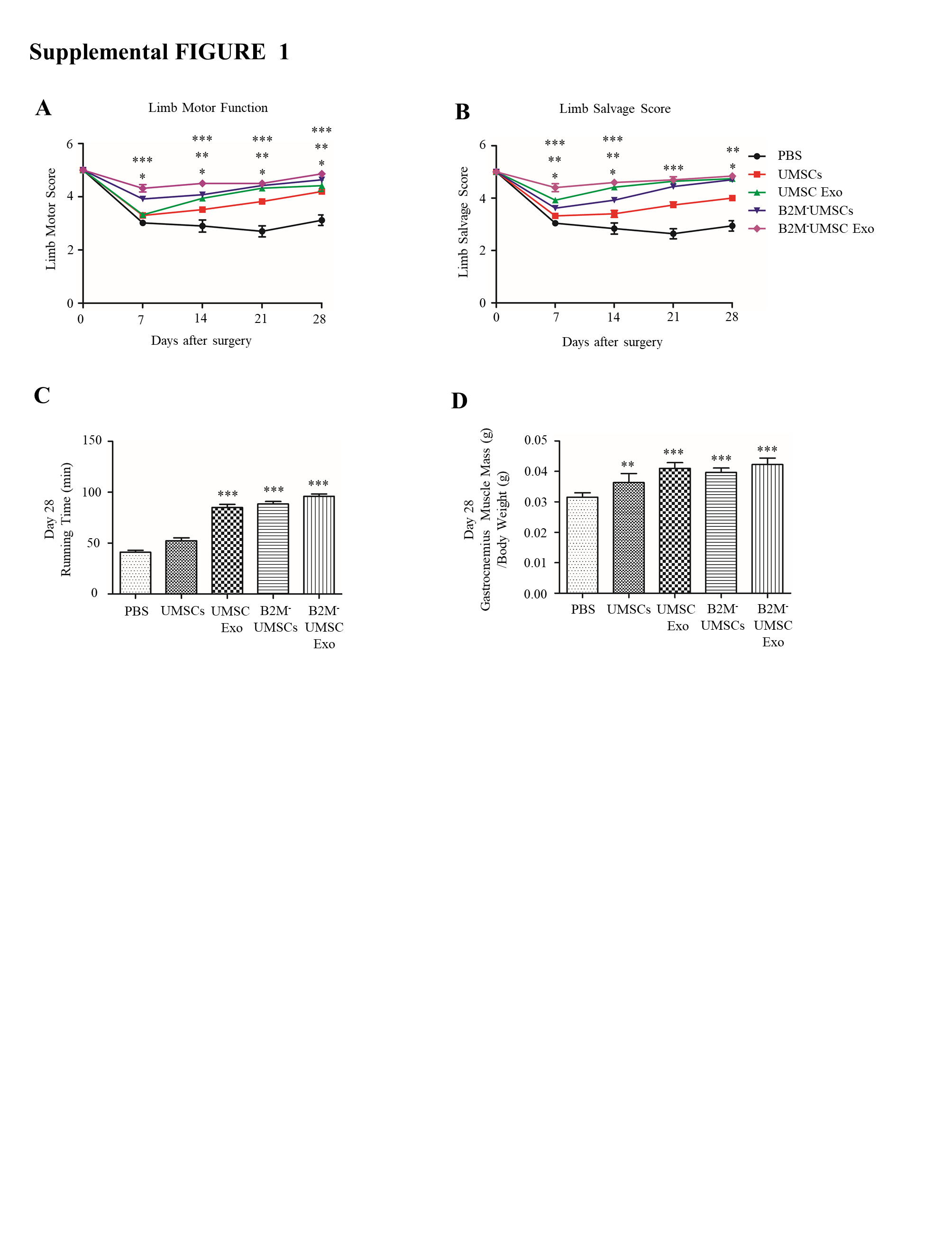
**

**FIGURE 1** B2M^-^UMSCs and exosomes improve functional recovery in the ischemic mouse hindlimb. (A) Mouse limb motor function. (B) Limb salvage. (C) Running time. (D) Muscle mass relative to body weight. N = 5/group. *P <0.05, **P <0.01, ***P <0.001.

**Supplemental Figure 2**

**
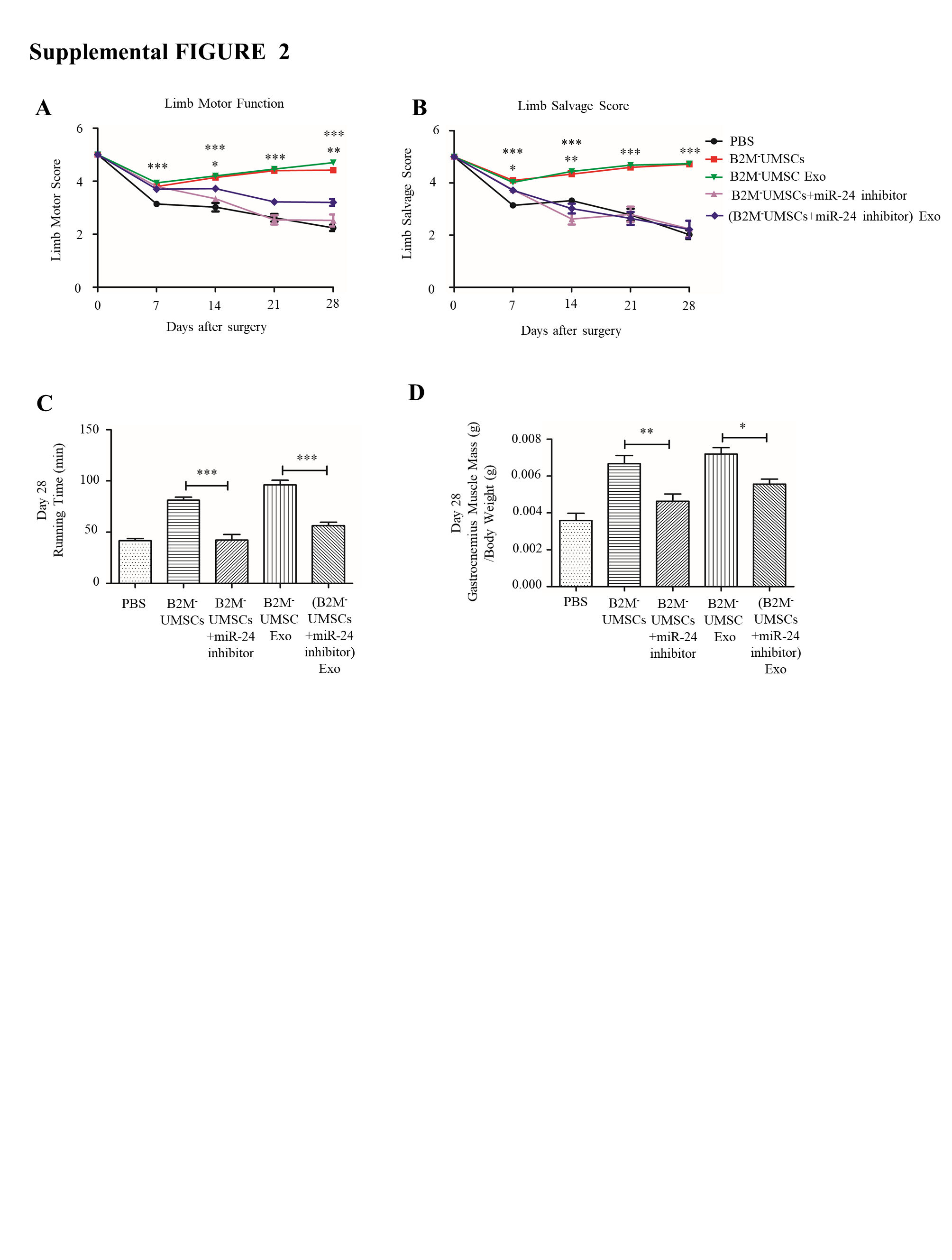
**

**FIGURE 2** miR-24 inhibitor blocks the improvement of functional recovery in the ischemic mouse hindlimb. (A) Mouse limb motor function. (B) Limb salvage. (C) Running time. (D) Muscle mass relative to body weight. N = 5/group. *P <0.05, **P <0.01, ***P <0.001.

**Supplemental Figure 3**


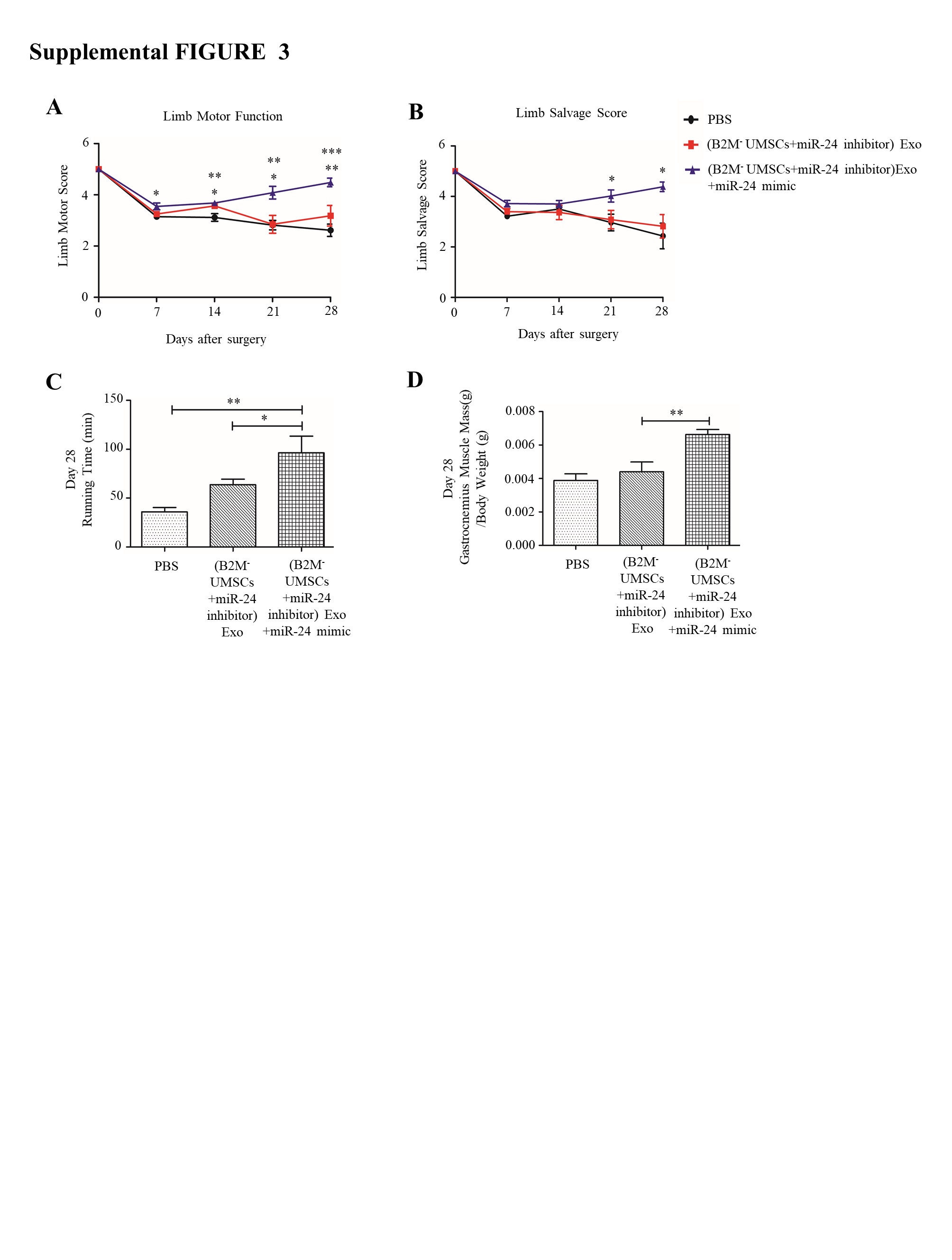


**FIGURE 3** miR-24 is necessary in improving functional recovery in the ischemic mouse hindlimb. (A) Mouse limb motor function. (B) Limb salvage. (C) Running time. (D) Muscle mass relative to body weight. N = 6/group. *P <0.05, **P <0.01, ***P <0.001.
